# SYSTEMATIC REVIEW AND BRAZILIAN EXPERT CONSENSUS ON THE USE OF BIOSIMILARS IN INFLAMMATORY BOWEL DISEASE: BRAZILIAN ORGANIZATION FOR CROHN’S AND COLITIS

**DOI:** 10.1590/S0004-2803.24612026-011

**Published:** 2026-10-05

**Authors:** Cristina FLORES, Francisco Guilherme Cancella PENNA, Marcello IMBRIZI, Adalberta Lima MARTINS, Renata de Sá Brito FRÓES, Munique Kurtz de MELLO, Stefania Burjack GABRIEL, Licia Maria RODRIGUES, Ligia Yukie SASSAKI, Marjorie Costa ARGOLLO, Fabio Vieira TEIXEIRA, Jane OBA, Eduardo Garcia VILELA, Rogério SAAD-HOSSNE, Matheus Freitas Cardoso de AZEVEDO, Júlio Maria Fonseca CHEBLI, José Miguel Luz PARENTE, Aderson Omar Mourão Cintra DAMIÃO, Eloá Marussi MORSOLETTO, Vera Lucia SDEPANIAN, Rogerio Serafim PARRA, Paulo Gustavo KOTZE

**Affiliations:** 1 DIIMUNO - Centro de tratamento de doenças inflamatórias intestinais e imunomediadas, Porto Alegre, RS, Brasil.; 2 Hospital das Clínicas da Universidade Federal de Minas Gerais, Belo Horizonte, MG, Brasil.; 3 University of Campinas, Faculty of Medical Sciences, Department of Internal Medicine, Division of Gastroenterology, Campinas, SP, Brasil.; 4 Private office, Vitória, ES, Brasil.; 5 Gastromed, Rio de Janeiro, RJ, Brasil.; 6 Universidade do Vale do Itajaí, Itajaí, SC, Brasil.; 7 Instituto Hospital de Base do Distrito Federal, Brasília, DF, Brasil.; 8 Universidade Federal do Maranhão, São Luís, MA, Brasil.; 9 Universidade Estadual Paulista (Unesp), Faculdade de Medicina, Botucatu, SP, Brasil.; 10 Universidade Federal de São Paulo, São Paulo, SP, Brasil.; 11 Gastrosaude, Marília, SP, Brasil.; 12 Universidade de São Paulo, São Paulo, SP, Brasil.; 13 Universidade Federal de Minas Gerais, Belo Horizonte, MG, Brasil.; 14 Universidade Federal de Juiz de Fora, Juiz de Fora, MG, Brasil.; 15 Universidade Federal do Piauí, Teresina, PI, Brasil.; 16 Hospital São Vicente, Curitiba, PR, Brasil.; 17 Faculdade de Medicina de Ribeirão Preto, Universidade de São Paulo, Ribeirão Preto, SP, Brasil.; 18 Pontifícia Universidade Católica do Paraná, Programa de Pós-Graduação em Ciências da Saúde, Curitiba, PR, Brasil.

**Keywords:** Biosimilar, anti-TNF, Inflammatory bowel disease, Crohn’s disease, Biossimilar, anti-TNF, Doença inflamatória intestinal, Doença de Crohn

## Abstract

**Background:**

Biologics improve outcomes in inflammatory bowel disease, but high costs restrict access and strain healthcare resources. Biosimilars lower costs, expand access, and support sustainable IBD care.

**Objective:**

Provide clear, evidence-based answers on biosimilar use in IBD across clinical, therapeutic, and economic domains to guide safe implementation and informed decision-making.

**Methods:**

GEDIIB conducted a PICO-structured systematic review across multiple databases and trial registries. We extracted clinically relevant outcomes and performed systematic review. We assessed risk of bias with RoB 2.0 and ROBINS-I and graded certainty using GRADE. A structured Delphi process with two voting rounds and external validation finalized recommendations, with consensus predefined at 85 percent or higher.

**Results:**

Anti-TNF biosimilars and originator biologics have similar efficacy, safety, immunogenicity, treatment persistence, and mucosal healing in biologic-naive patients and after switching, with no clinically meaningful differences in adverse events, loss of response, or discontinuation. Data on multiple switches do not indicate harm, though evidence remains limited. Pediatric studies suggest similar profiles, with lower precision. Economic evaluations show substantial cost savings and improved access, supporting the sustainability of the health system.

**Conclusion:**

Biosimilars represent a viable, safe, and cost-effective option for IBD management. We recommend structured switching strategies, clear communication with patients, and continuous clinical monitoring. Additional robust studies should strengthen evidence in specific populations and clinical scenarios.

## HIGHLIGHTS

• Biosimilars demonstrate efficacy and safety comparable to those of the originator in both inducing and maintaining remission. However, most supporting evidence derives from observational studies.

• Treatment and tracking protocols should utilize biosimilars in the same manner as biologic originators, including the assessment of serum levels and antibody monitoring.

• The adoption of biosimilars is associated with diminished healthcare costs while maintaining efficacy and without adversely affecting patient-reported outcomes or quality of life.

• The most debated aspects of biosimilar use include multiple switching between products and the nocebo effect. This article discusses the uncertainties regarding this subject.

## RESUMO

**Contexto:**

Os medicamentos biológicos melhoraram significativamente os desfechos clínicos na doença inflamatória intestinal (DII); entretanto, seus elevados custos limitam o acesso ao tratamento e impõem um impacto considerável aos sistemas de saúde. Os biossimilares reduzem custos, ampliam o acesso aos tratamentos e contribuem para a sustentabilidade da assistência aos pacientes com DII.

**Objetivo:**

Fornecer respostas claras e baseadas em evidências sobre o uso de biossimilares na DII, abrangendo aspectos clínicos, terapêuticos e econômicos, a fim de orientar sua implementação segura e subsidiar a tomada de decisões informadas.

**Métodos:**

A Organização Brasileira de Crohn e Colite (GEDIIB) conduziu uma revisão sistemática estruturada segundo a metodologia PICO, abrangendo múltiplas bases de dados e registros de ensaios clínicos. Foram extraídos desfechos clinicamente relevantes e realizada uma revisão sistemática. O risco de viés foi avaliado por meio das ferramentas RoB 2.0 e ROBINS-I, e a certeza da evidência foi classificada utilizando a metodologia GRADE. As recomendações foram elaboradas e validadas por meio de um processo Delphi estruturado, composto por duas rodadas de votação e de validação externa, tendo como consenso a concordância igual ou superior a 85%.

**Resultados:**

Os biossimilares anti-TNF e os biológicos originadores apresentam eficácia, segurança, imunogenicidade, persistência terapêutica e cicatrização mucosa semelhantes, tanto em pacientes virgens de tratamento biológico quanto após a troca do medicamento originador para o biossimilar. Não foram observadas diferenças clinicamente relevantes na ocorrência de eventos adversos, na perda de resposta ou na descontinuação do tratamento. Os dados disponíveis sobre múltiplas trocas não sugerem prejuízo clínico, embora as evidências permaneçam limitadas. Estudos pediátricos apontam perfis comparáveis de eficácia e segurança, porém com menor precisão das estimativas. Avaliações econômicas demonstram uma economia substancial de recursos e a ampliação do acesso ao tratamento, favorecendo a sustentabilidade dos sistemas de saúde.

**Conclusão:**

Os biossimilares representam uma alternativa viável, segura e custo-efetiva para o manejo da DII. Recomenda-se a adoção de estratégias estruturadas de troca terapêutica, de comunicação clara com os pacientes e de monitoramento clínico contínuo. Estudos adicionais, metodologicamente robustos, são necessários para fortalecer as evidências em populações específicas e em cenários clínicos particulares.

## INTRODUCTION

The advent of biologic therapies has significantly improved outcomes for patients with inflammatory bowel diseases (IBD). Beyond their clinical benefits, biologics have the potential to reduce the overall economic burden of IBD by decreasing indirect costs such as hospitalizations, surgeries, complications, and work productivity loss^1^ ^,^ ^2^. However, the high cost of originator biologics limits access and challenges the sustainability of the healthcare system. Biosimilars-biologic products that are highly similar to their originator agents in terms of quality, efficacy, and safety-represent an essential strategy to reduce treatment costs, increase accessibility, and promote long-term sustainability of IBD care^3^.

Accumulating evidence from systematic reviews and real-world studies demonstrates comparable efficacy, safety, and immunogenicity of anti-TNF biosimilars in both bio-naïve patients and after switching from the originator biologic^4^. The European Crohn’s and Colitis Organisation (ECCO), in its 2017 position statement, endorses the use of biosimilars in IBD management as a cost-effective approach that does not compromise clinical outcomes and supports switching when clinically appropriate^5^. Nevertheless, uncertainties remain regarding multiple switches between biosimilars, potential effects on immunogenicity, the nocebo effect, and the availability of data in specific subgroups, including pediatric patients and mucosal healing outcomes^6^ ^,^ ^7^. This expert consensus aims to provide clear, evidence-based answers to the most relevant questions on biosimilar use in IBD, addressing clinical, therapeutic, and cost-related aspects to support safe implementation and informed decision-making.

## METHODS

The GEDIIB (Brazilian Crohn’s and Colitis Organization) Guidelines on the Use of Biosimilars in Inflammatory Bowel Disease were developed through a formal and explicit systematic review structured around clinical questions formulated using the PICO framework (Population, Intervention, Comparator, and Outcomes). Eight clinical questions were predefined to address the main aspects of biosimilar use in IBD and to support the formulation of evidence-based recommendations.

The systematic review was conducted to identify and synthesize the best available evidence relevant to each PICO question. Scientific information sources included MEDLINE, Embase, Clinical Trials registries, Google Scholar, and manual searches of originator lists. Eligibility criteria, search strategies, and summaries of the included studies for each question are detailed in Annex 1. Extracted outcomes were clinically relevant endpoints rather than intermediate or surrogate measures and varied according to each clinical question. Whenever possible, results were expressed as absolute risks in each comparison group, risk differences with 95% confidence intervals, and numbers needed to treat (NNT) or harm (NNH).

The risk of bias of the included studies was assessed using the RoB 2.0 tool for randomized clinical trials and the ROBINS-I instrument for observational studies^8^-^9^. The overall quality of evidence was evaluated according to the GRADE methodology^10^ and classified as very low, low, or high, based on study design, risk of bias, and consistency of results, particularly when meta-analysis was not feasible.

When studies reported comparable outcomes, data were synthesized quantitatively through meta-analysis using appropriate statistical software. Analyses were conducted using fixed- or random-effects models, depending on the degree of heterogeneity, which was assessed using the I^2^ statistic. A summary of the evidence was generated for each clinical question to support the formulation of recommendations.

Following the systematic review, a Delphi consensus process was used to refine expert opinions and reach agreement on the recommendations. This structured and iterative method involved two rounds of anonymous voting with controlled feedback. Consensus was defined a priori as agreement of at least 85% among voting experts. Each PICO question, along with its corresponding evidence synthesis and proposed recommendation, was discussed by the expert panel, with consideration of efficacy, safety, and practical aspects of biosimilar use.

External validation was conducted throughout the process by a GEDIIB commission of specialists. In the first voting round, agreement rates for questions 1 through 8 were 100%, 100%, 91%, 100%, 96.6%, 100%, 91%, and 100%, respectively. A second online round was conducted to address dissenting opinions, resulting in revisions to selected recommendations. Final approval was obtained during an online consensus meeting, followed by a second round of voting, with agreement from all 23 participating experts.

The study selection process is presented in the PRISMA flow diagram (Figure 1) (Table 1), in accordance with PRISMA 2020 recommendations^11^.

**FIGURE 1:**
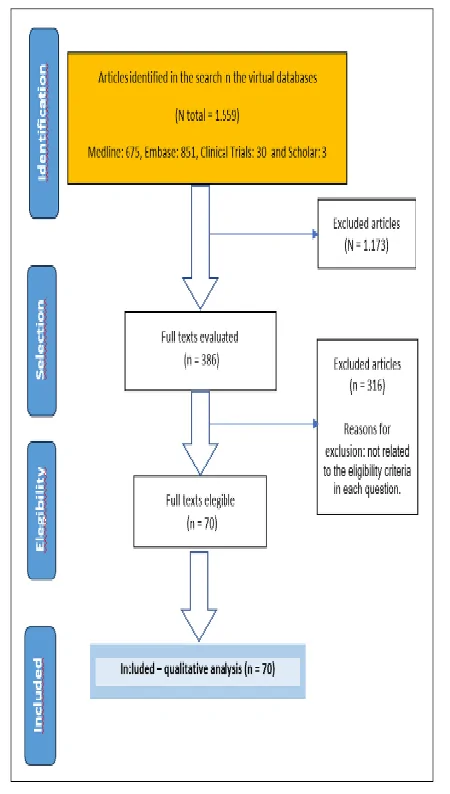
PRISMA flow diagram- Biosimilar in IBD Adults’ patients.

**TABLE 1:** Clinical Questions: statements, level of evidence, and agreement.

Questions	Statements	Quality of evidence	Level of Agreement
**1. In patients with IBD, what is the effect of treatment with biosimilar biologics compared to originator biologics on the induction of remission?**	Biosimilars are effective in maintenance and do not increase the risk of loss of response or discontinuation.	Low quality of evidence.	100%
**2. In patients with IBD, what is the effect on maintenance or discontinuation of treatment with biosimilar biologicals compared to the originator biologicals?**	Infliximab and adalimumab biosimilars are effective in maintenance and do not increase the risk of loss of response or discontinuation.	Very low quality of evidence.	100%
**3. In patients with IBD, what are the adverse effects of treatment with biosimilar biologics compared to the originator biologics?**	There is no difference in adverse effects between therapy with biosimilar and originator biologics in patients with IBD.	Low quality of evidence.	91%
**4. In patients with IBD and undergoing treatment with biosimilar biologicals, what is the role of the serum level of biological and anti-drug antibodies?**	In patients with IBD treated with biosimilar anti-TNF biologics, measuring serum levels and anti-drug antibodies can support decisions regarding dose escalation or reduction, or even change the drug treatment or regimen.	Low quality of evidence.	100%
**5. In patients with IBD, what is the effect on maintenance or discontinuation of treatment with biosimilar biologicals compared to the originator biologicals after multiple switches?**	The number of switches was not independently associated with persistence in the biological treatment, nor was it related to clinical remission or loss of response. Therefore, although the evidence is limited, multiple switches cannot be deemed ineffective or unsafe. Due to the evidence’s low quality, it is not possible to recommend for or against multiple switches.	Low quality of evidence.	95.6%
**6. In pediatric patients with IBD, what is the efficacy and safety of treatment with biosimilar biologics compared to the originator biologics?**	In pediatric patients with IBD, biosimilar biologics demonstrate efficacy and safety comparable to those of originator biologics; however, current evidence is insufficient to quantify these outcomes precisely.	Very low quality of evidence.	100%
**7. In patients with IBD, what is the impact on the cost of treatment with biosimilar biologics compared to the originator biologics?**	In patients with IBD, the use of biosimilar biologics for maintenance therapy is associated with lower treatment costs compared with originator biologics.	Low quality of evidence.	91%
**8. In patients with IBD, what is the effect on mucosal healing of treatment with biosimilar biologics compared to the originator biologics?**	In patients with IBD, treatment with biosimilar biologics achieves mucosal healing rates comparable to those with originator biologics. No significant differences are observed between switch and non-switch groups.	Low quality of evidence.	100%

## QUESTION 1

In patients with IBD, what is the effect of treatment with biosimilar biologics compared to originator biologics on the induction of remission?

### Practice point - Question 1

The use of biosimilars in treating IBDs (IBD) has been extensively studied, with evidence supporting their efficacy and safety comparable to those of originator biologics. Randomized controlled trials, such as the NOR-SWITCH study, demonstrated no significant difference in remission rates between patients who continued infliximab originator therapy and those who switched to the biosimilar CT-P13, confirming the non-inferiority of this approach^12^ ^,^ ^13^. Furthermore, large-scale observational cohort studies reinforce these findings, showing similar rates of clinical response and sustained remission between biosimilars and their originator biologics^14^ ^,^ ^15^.

Although observational studies inherently present a higher risk of bias, the consistency of clinical outcomes strengthens the recommendation that biosimilars are a viable and practical option for IBD treatment. No statistically significant differences in remission were observed between patients receiving the biosimilar and those on the originator in a persistence analysis of adalimumab biosimilars^16^. Additionally, multiple cohort studies have assessed non-medical switching between infliximab and adalimumab biosimilars, finding no evidence of worse clinical outcomes or increased loss of response over time^17^ ^,^ ^18^. Thus, a growing body of clinical evidence supports the recommendation that biosimilars effectively maintain remission and do not increase the loss of response or discontinuation rates. However, the quality of evidence remains low due to the predominance of observational studies.

#### Evidence Synthesis Table 2 ^12^ ^-^ ^35^.

**TABLE 2:** Evidence Synthesis Question 1: In patients with IBD, what is the effect of treatment with biosimilar biologics compared to the originator biologics on inducing remission?

First author	Year	Study design	N	Evidence Synthesis
Goll GL	2017	Randomised	380	Disease worsening occurred in 16.8% of the maintenance group and 11.6% of the switch group, with an adjusted risk difference of 5.9% (95%CI: -1.1 to 12.9), well within the noninferiority margin. Disease progression after 26 weeks was comparable between groups. By week 78, 61.1% of the maintenance group and 67.6% of the switch group achieved clinical remission, with an adjusted rate difference of -4.9% (95%CI: -13.4 to 3.7)^12^.
		OLE		
		NOR-SWITCH		
Jørgensen KK	2020	randomised, double-blind	248	NOR-SWITCH - 248 patients with IBD were randomized. 123 patients switched treatment from IFX to CT-P13, and 125 patients continued treatment with IFX. At the end of the main study (week 52), clinical remission was present in CD in 41 out of 63 patients (65%) and 46 out of 66 patients (70%) in the CT-P13 and IFX groups, respectively (adjusted risk difference 5.6%, 95%CI −11 to 22.2)^13^.
Chaparro M	2019	Observational, retrospective	476	Among 199 switch cohort (SC) patients, 38 relapsed after a median of 18 months; among 277 no switch cohort (NC) patients, 24 relapsed after 23 months. SC had a higher relapse rate (14% per patient-year) compared to NC (5% per patient-year) [*P*<0.05]. Cumulative relapse in the SC was 5% at 6 months, 14% at 12 months, and 28% at 24 months, versus 2%, 4%, and 10% in the NC over the same periods [*P*<0.05]^14^.
Haifer C	2021	Observacional prospective	204	Switching IBD patients from originator to biosimilar infliximab is safe and as effective as continuing the original treatment^15^.
Fernández-Cano MC	2024	Observacional retrospective	104	Adalimumab biosimilars show both safety and efficacy for IBD^16^
Lontai L	2022	Observacional prospective	276	There was no significant difference in clinical remission rates before and after switching from originator to biosimilar ADA (87.3% / 88.5% / 86.5% / 85.7%; *P*=0.888)^17^ ^,^ ^35^.
Martínez-Lozano H	2021	Observacional retrospective	239	The study suggests both the safety and efficacy of the double switch from originator IFX to CT-P13 and SB2 in patients with IBD is maintained^18^.
Bokemeyer B	2023	Observacional prospective	2543	The proportions of patients with remission by disease type (CD and
		Post-marketing		UC) for patients in the CT-P13 and IFX-RP groups were broadly similar to those for the overall IBD populations^5^ ^,^ ^19^.
Casanova MJ	2023	Observacional prospective	524	Relapse rates in the biosimilar group were 3% at 6 months, 6% at 12 months, and 26% at 24 months; in the originator group, rates were 3%, 6%, and 12% at these time points (*P*=0.09)^20^.
Barberio B	2021	Observacional prospective	156	Clinical benefit was observed in 85.3% of patients (116/155). Rates were 88.7% for ADA, 86.3% for ABP501, and 79.5% for SB5; there was no significant difference between these drugs^21^.
Cingolani L	2021	Observacional prospective	55	Kaplan-Meier curves showed no significant efficacy difference between patients switching to ABP501 and those continuing the ADA originator (*P*=0.20)^22^
Ho SL	2020	Observacional retrospective	2818	The authors used propensity score matching to assess non-inferiority of the IFX biosimilar. Non-inferiority was confirmed, as the −7.2% difference in disease worsening requiring acute care met the +10% margin. Patients who switched to infliximab-dyyb had a significantly lower risk of disease worsening than those who stayed on RP infliximab (*P*<0.01)^23^.
Farkas K,	2016	Observacional prospective	63	At week 14, 82.5% of CT-P13 patients achieved cumulative clinical response and 47.6% reached steroid-free remission^24^.
Kaniewska M	2021	Observacional retrospective	168	There was no significant difference in remission rates between groups: IFX - 80.9% (naïve) vs 79.4% (non-naïve), *P*=0.85; BIFX-73.1% vs 74.1%, *P*=0.93^25^.
Kumar P	2022	Observacional retrospective	137	After 52 weeks, 57.1% of CD patients on originator and 68.4% on biosimilar maintained clinical response (OR: 1.62; *P*=0.380). For UC, 66.7% with originator and 68.7% with biosimilar maintained response (*P*=0.88)^26^.
Luber RP	2021	Observacional prospective	186	After one year, patients with stable infliximab dosing showed no significant change in disease activity scores. The proportion with clinical disease activity (HBI or SCCAI ≥5) remained similar to before the switch (5.0% vs 9.1%, *P*=0.16)^27^.
Lukas M	2022	Observacional prospective	54	Mean HBI scores were below 5 in both biosimilar and originator groups at all time points, with no clinically meaningful differences between cohorts^28^.
Lukas M	2020	Observacional prospective	186	SB2 appears to have safety and effectiveness comparable to IFX originator and CT-P13 ^29^.
McClinchie MG	2023	Observacional retrospective	152	At week 22, clinical remission was seen in 13 patients (81%) on the originator and 5 patients (19%) on the biosimilar (*P*=0.02)41^30^.
Martín-Gutiérrez N	2022	Observacional prospective	42	Long-term real-world data show that Originator and Biosimilar IFX CTP-13 have similar efficacy and safety in IBD patients^31^.
Meyer A	2019	Observational, retrospective	3112	There was no significant difference in effectiveness between the RP and CT-P13 groups (*P*=0.20). At 12 and 24 months, cumulative incidence rates were 43.0% and 57.5% for RP, and 45.1% and 59.8% for CT-P13, respectively^32^.
Meyer A	2019	Observacional retrospective	5050	Clinical remission occurred in 82.3% of GP2017 patients and 75.0% of those on ADA originator (*P*=0.311)^33^.
Serrano Díaz L	2024	Observacional retrospective	153	Demonstrates comparable efficacy between both drugs (biosimilar and originator) in a real clinical setting^34^ ^,^ ^64^
Valcuende-Rosique A	2022	Observacional retrospective	80	There was no significant change in the rates of clinical remission (CR) or corticosteroid-free clinical remission (CSF-CR) at 8 weeks pre-switch, baseline, week 12, or week 24 after switching to biosimilar68 (*P*=0.129 for CR; *P*=0.113 for CSF-CR)^35^.

#### Recommendations - Question 1

Biosimilars are effective in inducing remission and do not increase the risk of loss of response or discontinuation.

Infliximab and adalimumab biosimilars are effective in inducing remission and do not increase the risk of loss of response or discontinuation.

Low quality of evidence.

## QUESTION 2

In patients with IBD, what is the effect on maintenance or discontinuation of treatment with biosimilar biologicals compared to the originator biologics?

### Practice point - Question 2

The evidence on the maintenance and discontinuation of biosimilar biologics versus originator biologics in patients with IBD suggests comparable persistence rates. While randomized controlled trials are limited, available observational cohort studies indicate that switching from originator to biosimilars does not result in increased rates of treatment discontinuation due to loss of response or adverse events^16^ ^,^ ^36^. Data from prospective and retrospective studies suggest that persistence with biosimilars is similar to, or slightly lower than, that with originator biologics, without a significant impact on clinical outcomes^20^ ^,^ ^37^. Furthermore, switching from biosimilar to biosimilar has demonstrated comparable persistence rates, reinforcing the concept of interchangeability in clinical practice^17^.

Although some studies indicate a higher rate of re-transitioning to the originator after switching to a biosimilar, the primary reasons for discontinuation remain consistent across treatment groups, predominantly due to loss of response or adverse effects^37^ ^,^ ^38^. The NOR-SWITCH trial and its extension further support the safety and efficacy of biosimilar switching, demonstrating no significant differences in treatment persistence between patients who continued infliximab originator and those who switched to CT-P13^39^. However, the quality of the evidence remains very low, primarily because of the predominance of observational studies with a high risk of bias. Despite these limitations, the available data support the recommendation that biosimilars are effective for maintenance therapy and do not increase the risk of treatment discontinuation.

Evidence Syntheses Table 3 ^14^ ^-^ ^22^ ^,^ ^26^ ^-^ ^28^ ^,^ ^31^ ^,^ ^33^ ^,^ ^36^ ^-^ ^48^.

**TABLE 3:** Evidence Synthesis Question 2: In patients with IBD, what is the effect on maintenance or discontinuation of treatment with biosimilar biologics compared to the originator biologics?

First author	Year	Study design	N	Evidence Synthesis
Chaparro M	2019	Observational, retrospective	476	In the multivariate analysis, the switch to CT-P13 was associated with a higher risk of relapse (HR=3.5, 95% confidence interval [CI] = 2-6). Thirteen percent of patients had adverse events in the NC, compared with 6% in the SC [*P*<0.05]. Switching from Remicade to CT-P13 may be associated with a higher risk of clinical relapses; however, this was not supported by an increase in objective markers of inflammation in our study^14^.
Haifer C	2021	Observacional prospective	345	Infliximab therapy was intensified or discontinued for 31 patients receiving CT-P13 (15%) and 20 patients receiving originator infliximab (14%) (adjusted HR, 1.39; 95%CI, 0.71-2.76)^15^.
Fernández-Cano MC	2024	Observacional retrospective	104	During follow-up, four patients (8%) on biosimilars, 11 (38%) on originators, and 9 (36%) who switched from biosimilars to originators discontinued treatment^16^.
Lontai L	2022	Observacional prospective	276	Patients who switched from the originator to a biosimilar ADA had a probability of 95.4% (SE:1.6) to remain on medication after 20 weeks, and 91.6% (SE: 2.2) after 40 weeks^17^.
Martínez-Lozano H	2021	Observacional retrospective	239	Of the 153 patients with CD, O-IFX was initiated in 95 patients and B-IFX in 58. The mean duration of IFX exposure was 30.58 (18.44) months for O-IFX and 17.15 (10.09) months for B-IFX. The persistence rates of IFX treatment were not statistically significant, as determined by the log-rank test^18^.
Bokemeyer B	2023	Observacional prospective	2543	In the IFX-RP cohort, 23.7% of patients discontinued from treatment, primarily due to AEs. In the CT-P13 group, 38.9% of patients discontinued from treatment, mainly due to AEs^19^.
		Post-marketing		
Casanova MJ	2023	Observacional prospective	524	The cumulative incidence of ADA discontinuation in the biosimilar was 0% at 6 months and 4% at 12 months, whereas the corresponding rates for the originator were 1% and 4%, respectively (*P*>0.05)^20^.
Barberio B	2021	Observacional prospective	155	In this multicenter cohort, 35.5% of patients started on ADA originator, 34.8% on ABP501, and 29.7% on SB5 biosimilars. Seventeen discontinued therapies (6 ADA, 7 ABP501, 4 SB5; *P*=0.30), with five due to adverse events (2 ADA, 2 ABP501, 1 SB5) and 12 for inefficacy (4 ADA, 5 ABP501, 3 SB5)^21^.
Cingolani L	2021	Observacional prospective	55	Kaplan-Meier curves showed no significant efficacy difference between patients switching to ABP501 and those continuing the ADA originator (*P*=0.20)^22^.
Kumar P	2022	Observacional retrospective	137	In the originator group, treatment was discontinued at 52 weeks for 51 of 102 patients (50%). Among 35 biosimilar patients, 13 (37%) discontinued early-11 due to loss of response and two due to adverse effects^26^.
Luber RP	2021	Observacional prospective	186	Clinical remission rates remained stable at both early (92% vs 91%, *P*=0.75) and one-year (95% vs 91%, *P*=0.16) assessments. The time to loss of response did not differ significantly between first-time and second-time switchers (*P*=0.69)^27^.
Lukas M	2022	Observacional prospective	54	Over the entire follow-up period (up to 104 weeks), persistence on SB5-adalimumab therapy was statistically significantly lower versus originator-adalimumab (stratified log-rank *P*<.001)^28^.
Martín-Gutiérrez N	2022	Observacional prospective	42	Thirty-six (85.7%) of patients remained on CT-P13 at the 2-year follow-up. Two patients discontinued CT-P13 due to loss of response, despite having adequate serum infliximab concentrations (4.8% of the total at baseline). Four patients did not complete the full 2-year post-switch follow-up due to nonadherence (n=2), pregnancy (n=1), or transfer to another healthcare center (n=1)^31^.
Meyer A	2019	Observational, retrospective	3112	208 (14.5%) patients discontinued infliximab in the RP group and 187 (11.1%) patients discontinued infliximab in the CT-P13 group; in addition, 163 (11.4%) and 171 (10.2%) patients, respectively, switched to the other form of infliximab^32^.
Meyer A	2019	Observational, retrospective	5050	During follow-up, 18.6% of patients discontinued infliximab in the RP group, 17.6% of patients discontinued infliximab in the CT-P13 group, and 6.3% and 8.2% of patients, respectively, switched to the other form of infliximab48^33^
Wetwittayakhlang P	2024	Observacional prospective	210	Patients who underwent a biosimilar switch from an IFX- or adalimumab-originator had a similar probability of drug persistence. (Log-rank = 0.793)^36^
Meijboom RW	2023	Retrospective Observacional prospective	575	In this real-world study of patients with IBD who were on biosimilar infliximab and then retransitioned to originator infliximab vs. those who remained on biosimilar, a total of 575 patients were included. Patients who retransitioned did so mainly due to perceived loss of effect or adverse events. Discontinuation rates were similar between the retransitioned and biosimilar-continuing groups, indicating no increased risk of discontinuation after switching back to the originator.
Hellström PM	2022	Observacional prospective	216	IFX treatment was stable (uninterrupted for a period of 120 days between infusions during follow-up) among 48% of switchers and 78% of non-switchers.
Jørgensen KK	2020	randomised, double-blind	248	Throughout the main study, 13 CD patients discontinued the study: six in the CT-P13 group and seven in the IFX group. In the UC group, six patients discontinued participation: one in the CT-P13 group and five in the IFX group. There weren’t statistically significant differences.
Barberio B	2020	Observational, retrospective	179	At week 52, 31.1% of ADA, 16.7% of IFX originator, and 36.2% of biosimilar patients needed treatment optimization. At week 52, 13 patients had suspended therapy because of severe adverse events, including 3 cases of malignant disease. Anti-TNF-α treatment was more effective in patients with CD than in those with UC, regardless of the drug used^40^.
Lin I	2022	Observacional retrospective	1687	Patients with CD, RA, PsA, PsO, and UC. During an average follow-up of 713 days for switchers and 464 days for continuers, 82.6% of switchers and 35.0% of continuers stopped their index therapy (HR=3.12 [2.66, 3.66]; *P*<.001)^41^.
Iniesta NC	2021	Observacional retrospective	106	At week 54, treatment was withdrawn in 15.1 % of patients (11.5 % for the originator IFX and 20.0 % for CPT-13, *P*=0.226), and in 27.4 % of patients at week 104 (26.2 % for the originator IFX and 28.9 % for CPT-13, *P*=0.761)26^42^.
Martínez-Feito A	2020	Observational, retrospective and prospective	86	Patients in the infliximab-O group were analyzed retrospectively, while those receiving the biosimilar were followed prospectively until week 22. None of the originators withdrew; however, 7 biosimilar patients (3 CD, 4 UC) discontinued at week 22: 3 due to secondary failure, 3 due to primary failure, and 1 due to surgery^43^.
Ratnakumaran R	2018	Observacional prospective	210	At 12 months following the switch to CT-P13, 146 (76.4%) patients maintained their response to infliximab, compared with twelve (63.2%) patients who continued on the infliximab originator (*P*=.20).
Sagami S	2022	Observacional prospective	640	The 1-year persistence rates for CT-P13 and its originator were the same for both CD (84%) and UC (70%).
Serrano Díaz L	2024	Observacional retrospective	153	At week 26, treatment was withdrawn in 21.6% of patients (28.6% CPT-13 and 17.2% originator IFX, *P*=0.102), and at week 52, treatment was withdrawn in 25.5% of patients (31.7% CPT-13 and 21.5% originator IFX, *P*=0.159).
Smith JT	2021	Observacional retrospective	3206	Among bionaïve patients, 870 (27.1%) initiated infliximab-dyyb, and 2336 (72.9%) started infliximab; baseline characteristics differed between groups. Switching to another biologic was more frequent in the infliximab-dyyb group (21.0%) than in the infliximab group (15.2%; HR 1.55, CI 1.30-1.85, *P*<.01).
Trystam N	2021	Observacional prospective	158	Double switching from the originator Infliximab to CT-P13 and then to SB2 was not associated with an impairment in patient beliefs, while the effectiveness, immunogenicity, and safety of anti-TNF therapy remained stable after 54 weeks of follow-up.

#### Recommendations - Question 2

Biosimilars are effective in maintenance and do not increase the risk of loss of response or discontinuation.

Very low quality of evidence.

## QUESTION 3

In patients with IBD, what are the adverse effects of treatment with biosimilar biologics compared to the originator biologics?

### Practice point - Question 3

Several comparative studies, including randomized clinical trials and real-world data, demonstrate that biosimilars of biologic agents used in the treatment of IBD (such as infliximab and adalimumab) have safety profiles comparable to those of their originators.

The rates of adverse events, including infusion reactions, infections, immunogenicity (such as the development of anti-drug antibodies), and serious events, do not differ significantly between biosimilars and originators.

In addition, switching studies-in which patients stable on originators were switched to biosimilars-have shown no consistent increase in adverse events, reinforcing the safety of switching in clinical practice.

#### Evidence Syntheses Table 4 ^12^ ^-^ ^14^ ^,^ ^16^ ^,^ ^18^ ^,^ ^21^ ^,^ ^26^ ^,^ ^27^ ^,^ ^32^ ^,^ ^33^ ^,^ ^36^ ^,^ ^44^ ^,^ ^45^ ^,^ ^47^ ^-^ ^57^.

**TABLE 4:** Evidence Synthesis Question 3: In patients with IBD, what are the adverse effects of treatment with biosimilar biologics compared to the originator biologics?

First author	Year	Study design	N	Evidence Synthesis
Goll GL	2017	Randomised Open label extension	380	The 26-week NOR-Switch extension study included 380 CT-P13 users. Adverse events were reported by 44% in the maintenance group and 40% in the switch group, with no difference in infusion reactions between the two arms^12^.
Jørgensen KK	2017	randomised, double-blind	481	Similar numbers of patients had at least one treatment-emergent adverse event in the two treatment groups (168 [70%] in the infliximab originator group and 164 [68%] in the CT-P13 group) ^13^.
Chaparro M	2019	Observational, retrospective	476	A total of 48 patients developed 61 adverse events during follow-up. The proportion of patients that developed adverse events was 13% in the NS and 6% in the SC [*P*<0.05]^15^
Fernández-Cano MC	2024	Observacional retrospective	104	During follow-up, 4 patients (8%) on biosimilars, 11 (38%) on originators, and 9 (36%) who switched from biosimilars to originators discontinued treatment^16^.
Martínez-Lozano H	2021	Observacional retrospective	239	At week 14, two (2.1 %) SAEs (infusion reactions) were registered in CD patients with O-IFX and five (8.6 %) CD patients treated with B-IFX experienced SAES (*P*=0.105). Among UC patients, the mean duration of IFX exposure was also notably longer in the O-IFX cohort than in the B-IFX cohort. A total of four (10%) subjects in the O-IFX group and five (10.9 %) subjects in the B-IFX group had SAEs during the study^18^.
Bokemeyer B	2023	Observacional prospective	2543	In the IFX-RP cohort, 23.7% of patients discontinued from treatment, primarily due to AEs. In the CT-P13 group, 38.9% of patients discontinued from treatment, due to AEs^19^.
Casanova MJ	2023	Observacional retrospective	524	A total of 79 patients reported 95 adverse events during follow-up. The proportion of patients who developed adverse events related to ADA was 6% in the SC and 5% in the NSC (*P*>0.05). There was no statistically significant difference between the originator and the biosimilar^20^.
Barberio B	2021	Observacional prospective	156	10 patients stopped therapy because of AEs, without statistically significant difference between the groups (*P*=0.65)^21^.
Kumar P	2022	Observacional retrospective	137	In the originator group, treatment was discontinued at 52 weeks for 51 of 102 patients (50%). Among 35 biosimilar patients, 13 (37%) discontinued early-11 due to loss of response and 2 due to adverse effects^26^.
Jørgensen KK	2020	randomised, double-blind	248	123 patients switched treatment from IFX to CT-P13, and 125 patients continued treatment with IFX. Similar numbers of patients had at least one treatment-emergent AE in the two treatment groups in CD and UC in the main and extension study^27^.
Meyer A	2019	Observational, retrospective	3112	A total of 157 (47.6/1000 PY) serious infections were identified. Multivariable analysis demonstrated fewer serious infections in the CT-P13 group (HR 0.65; 95%CI: 0.48-0.88)^32^.
Meyer A	2019	Observational, retrospective	5050	2551 (50.5%) in the RP group and 2499 (49.5%) in the CTP13 group. Multivariable analysis did not demonstrate any significant differences between RP and CT-P13 in serious infections (HR, 0.82 [CI, 0.61 to 1.11])^33^.
Wetwittayakhlang P	2024	Observacional prospective	210	Patients who underwent a biosimilar switch from an IFX- or adalimumab- originator had a similar probability of drug persistence. (Log-rank = 0.793)^36^.
Ratnakumaran R	2018	Observacional prospective	210	Adverse events occurred in 9 (4.7%) patients who switched to CT-P13, but in none of those who continued the originator infliximab *(P*=1.0)^44^.
Sagami S	2022	Observacional prospective	640	This is a post-market surveillance. There was no significant difference in the adverse events between patients treated with CT-P13 and patients treated with the originator IFX^45^.
Smith JT	2021	Observacional retrospective	3206	There were no statistically significant differences in the risks for any infections (HR 1.01; 95%CI 0.86-1.17; *P*=.95), serious infections (HR 0.83; 95%CI 0.54-1.26; *P*=.38), cancers (HR 0.83; 95%CI 0.44-1.54; *P*=.55), or tuberculosis (HR 0.59; 95%CI 0.10-3.55; *P*=.57)^47^.
Trystam N	2021	Observacional prospective	158	Adverse events occurred in 63 (39.9%) patients including 50 (41.1%) in the double switch group and 13 (31.6%) in the single switch group (*P*=0.15), accounting for 77 adverse events and 10 serious adverse events (6.3%). There was a higher rate of infectious adverse events (0.40±0.60 vs 0.16±0.43, *P*=0.02) in the double switch group^48^.
Gros B	2023	Observacional prospective	297	Six adverse events occurred in five patients: psoriatic reaction, tonsil squamous cell carcinoma, arthralgia, severe COVID infection needing ICU care, heart failure with ICU admission, and mild skin reaction. Three were severe enough to require discontinuing the drug. There was no predominance of side effects in patients using the biosimilar compared to the originator^49^.
Hanauer S	2021	Clinical trial, randomised	147	In the safety analysis set, 45 (63%) of 72 patients in the BI 695501 group and 42 (56%) of 75 in the adalimumab originator product group had an adverse event during weeks 0-24; 31 (43%) and 34 (45%) had adverse events during weeks 24-56^50^.
Mazza S	2022	Observacional retrospective	52	No differences in the IFX discontinuation rates due to AEs over time were observed between double-switched and single-switched patients. Moreover, no differences in the frequency of overall and type-specific AEs were found between the double-switch and single-switch strategy^51^.
Rusch C	2022	Observacional retrospective	188	There were no differences in the incidence of infusion reactions among rapid infusion infliximab, and infliximab biosimilars (*P*=0.863)^52^.
Mocci G	2022	Observacional retrospective	134	AEs occurred in 2 UC patients (4.9%) and 3 CD patients (3.2%). Treatment discontinuation due to AEs was reported in two ADA originator patients and one GP2017 patient, with no significant difference between groups^53^.
Macaluso FS	2021	Observacional prospective	276	SB2 was used across initiation and switch scenarios with low combination immunomodulator use. In routine IBD care, SB2 appears safe for initiation and switching from originator IFX or CT P13, including for dose/interval optimization^54^.
Petitdidier N	2020	Observacional retrospective	364	We conducted a multicenter, retrospective, observational, usual care cohort study-all consecutive patients starting CT-P13 for active IBD. Adverse events occurred in 93 (25.5%) out of 364 patients. Serious adverse events occurred in 39 (10.7%) patients and were related to IBD exacerbation in 23 cases^55^.
Ye BD	2019	randomised, double-blind	220	During the 1-year study, 147 patients (67%) reported at least one treatment-emergent adverse event: 36 (64%) in CT-P13-CT-P13, 34 (62%) in CT-P13-infliximab, 37 (69%) in infliximab-infliximab, and 40 (73%) in infliximab-CT-P13 groups^56^.
Guerra Veloz MF	2018	Observational retrospective - prospective	98	Each cohort included 98 patients. A retrospective cohort treated with the originator IFX and a prospective cohort in which patients switched from the originator to the biosimilar CT-P13. There were no statistical differences between each group (*P*=0.814, 95%CI: 0.115-0.075)^57^.

#### Recommendations - Question 3

There is no difference in adverse effects comparing therapy with biosimilar and originator biologics in patients with IBD.

Low quality of evidence.

## QUESTION 4

In patients with IBD and undergoing treatment with biosimilar biologicals, what is the role of the serum level of the biological and anti-drug antibodies?

### Practice point - Question 4

Monitoring serum levels of biological drugs, particularly TNF antagonists (anti-TNF), is clinically relevant since subtherapeutic levels are often associated with loss of therapeutic response. One mechanism that reduces serum levels is the formation of anti-drug antibodies (ADA). Therefore, serum measurement of both the drug and anti-drug antibodies is justified as an important tool for monitoring treatment. Most studies show comparable Trough levels, ADA rates, and clinical activity for biosimilars versus originators; switching rarely alters these parameters meaningfully^13^ ^,^ ^15^ ^,^ ^28^ ^,^ ^34^ ^,^ ^36^ ^,^ ^39^ ^,^ ^42^ ^,^ ^58^.

Use TDM to differentiate low trough levels from immunogenicity and to guide both escalation and de-escalation; iterative optimization is frequently needed and should be performed^31^ ^,^ ^59^ ^,^ ^60^. Absence of antibodies and therapeutic TLs favors remission, but some patients lose response despite high TLs, signaling pharmacodynamic failure and the need to reassess the mechanism or therapeutic class^55^ ^,^ ^61^ ^,^ ^62^. For both IFX and ADL, antibody formation rates did not differ significantly between biosimilars and originators.

Outlier findings (such as higher trough levels of originator biologics during the early weeks of treatment) should be interpreted with caution, as they may reflect heterogeneity in study design, induction regimens, sampling schedules, and laboratory assays rather than actual differences between products^15^ ^,^ ^28^ ^,^ ^34^ ^,^ ^42^ ^,^ ^43^. Additionally, the interpretation of these results is limited by the relatively short follow-up periods in most studies, generally less than 1 year, and by the scarcity of randomized trials with greater methodological robustness^15^ ^,^ ^28^ ^,^ ^34^ ^,^ ^42^ ^,^ ^43^.

Evidence Synthesis Table 5 ^13^ ^,^ ^15^ ^,^ ^16^ ^,^ ^28^ ^,^ ^29^ ^,^ ^31^ ^,^ ^34^ ^,^ ^36^ ^,^ ^39^ ^,^ ^42^ ^-^ ^44^ ^,^ ^55^ ^,^ ^56^ ^,^ ^58^ ^-^ ^62^.

**TABLE 5:** Evidence Synthesis Question 4: In patients with IBD and undergoing treatment with biosimilar biologics, what is the role of the serum level of the biological and anti-drug antibodies?

First author	Year	Study design	N	Evidence Synthesis
Jørgensen KK	2017	randomised, double-blind	481	Trough drug concentrations were comparable in both groups during follow-up. ADAbs appeared in 11% of infliximab originator patients and 13% of CT-P13 patients. Excluding those with baseline ADAb, incidence rates were 7% for infliximab originator and 8% for CT-P13^13^.
Heifer C	2020	Observacional prospective	345	Infliximab trough levels were similar for the two groups at all time points, as were the proportions of patients with detectable anti-infliximab antibodies (originator infliximab, 12 [8.5%]; CT-P13, 15 [7.4%]). Clinical disease activity was also comparable for the two groups throughout the study^15^.
Fernández-Cano MC	2024	Observacional retrospective	104	During follow-up, 51.9% of patients had their serum adalimumab levels and antibodies checked. Of these, 72.2% showed adequate or high drug levels, while 27.8% had low levels. Anti-adalimumab antibodies were detected in only 5.5% of patients. Two of these patients were in the originator adalimumab group, and one was in the switched group^16^.
Lukas M	2022	Observacional prospective	186	Mean drug concentrations were similar between the originator-adalimumab and SB5-adalimumab cohorts at baseline, week 26, and week 52, with no clinically significant differences observed up to week 52^28^.
Lukas M	2020	Observacional prospective	186	Adalimumab trough levels remained stable from week 0 to week 10 in both the SWITCH (14.2 to 13.0 μg/mL) and ORIGINATOR cohorts (14.8 to 13.7 μg/mL), with no significant changes. At week 10, ADA positivity was 2% in both groups^29^.
Martín-Gutiérrez N	2022	Observacional prospective	42	Switching from the originator infliximab to the biosimilar CT-P13 for the treatment of inflammatory bowel disease (IBD) did not adversely affect long-term clinical outcomes or pharmacokinetics in patients. No comparative group^31^.
Park J	2023	Randomizado, duplo cego	198	Infliximab trough levels in responders in the CT-P13 group were not statistically different from those in the infliximab groups (*P*>0.05). The ADA positive rates were not different between CT-P13 and originator infliximab groups at week 14 (15.0% vs 19.4%, *P*=0.453).
Wetwittayakhlang P	2024	Observacional prospective	210	Patient proportions maintaining therapeutic drug levels were similar at all timepoints before and after the switch (ranging from 83.0% to 85.3%; *P*=0.597). ADAs developed in 2.5% of ADAL-treated patients (3 of 118), while high- and low-titer IFX-ADAs occurred in 2.2% and 7.6% of IFX-treated patients, respectively. Over 24 weeks, 11 patients (5.2%) required dose escalation, and 1 patient had therapy reduced.
Jørgensen KK	2020	randomised, double-blind	248	During the main study, new ADAbs appeared in 5% of CD patients (CT-P13) and 4% (IFX); in UC, 17% (CT-P13) and 11% (IFX). In the extension, no CD patients on CT-P13 maintenance and 1.6% switching from IFX developed ADAbs; for UC, rates were 2.4% (maintenance) and 2.6% (switch). Trough drug levels were similar across groups^39^.
Iniesta Navalón C	2021	Observacional retrospective	106	The pharmacokinetic characteristics and incidence of immunogenicity of CPT-13 in a real clinical setting are comparable between IFX originator and CPT-13.
Martínez-Feito	2020	Observational retrospective/prospective	86	IFX Originator trough levels were higher than the biosimilar trough levels (35 vs 21, 20.1 vs 11, 6.6 vs 2.9, and 4.3 vs 1.7 μg/mL at weeks 2, 6, 14, and 22, respectively). By week 22, 81.2% of patients in clinical remission received the originator treatment, whereas 18.8% were given the biosimilar (*P*=0.02)^43^.
Ratnakumaran R	2018	Observacional prospective	210	Patients who had therapeutic drug levels and no significant antibodies before switching were more likely to be in clinical remission at 12 months than those with low drug levels and relevant antibodies (66.7% vs 20%, *P*=.004)^44^.
Petitdidier N	2020	Observacional retrospective	364	The rate of patients achieving steroid-free clinical remission was higher in patients with the absence of ACA compared with those with the presence of ACA (65.7% vs 47.2%, *P*=0.01)^55^.
Ye BD	2019	randomised, double-blind	220	The proportion of patients with a positive ADA result was similar between the CT-P13 and infliximab treatment groups at week 14 (15 [14%] vs 19 [17%]) and week 30 (43 [39%] vs 49 [45%]). %]). The proportion who were positive for NAbs was also similar between groups at week 14 (10 [9%] vs 13 [12%]) and week 30 (22 [20%] vs 21 [19%])^56^.
Eberl A	2017	Observacional prospective	62	Sixty-two IBD patients switched from originator to biosimilar IFX for maintenance therapy. Median IFX trough levels remained unchanged at 5.5 mg/l before and after the switch (difference: 0.45 mg/l; *P*=0.05), indicating no significant change^58^.
Elosua González A	2020	Observacional prospective	77	This study compared CT-P13 trough levels between a clinical (CLN) and a proactive (PRO) group. Among patients in clinical remission at week 14, 13 PRO patients were intensified due to low drug levels. Secondary loss of response did not differ significantly between groups (PRO 0%, CLN 8.7%), nor did secondary response loss at week 54 (PRO 0%, CLN 8.7%; *P*=0.22). No cases of antibody-related loss of response occurred^59^.
Pekala A	2021	Observacional prospective	65	Patients with a response to induction treatment had a significantly higher average drug level than patients with primary lack of response (*P*=0.001) or with secondary loss of response (*P*<0.001). The incidence of ADA in patients with non-therapeutic drug levels was 9/44 (20.4%), while in patients with undetectable drug levels, it was 8/16 (50%).
Pekala A	2021	Observacional prospective	84	There was also a high rate of LOR among patients (CT-P13) with the highest drug levels: 50% in CD and 25% in UC (*P*=0.400).
Roblin X	2022	Observacional prospective	20	In patients with inactive CD under maintenance therapy with CT-P13 sc, therapeutic drug monitoring of IFX can be performed at any time between two CT-P13 sc injections.

#### Recommendations - Question 4

In patients with IBD treated with biosimilar anti-TNF biologics, measuring serum levels and anti-drug antibodies can support decisions on dose escalation or reduction, or even change the drug treatment or regimen.

Low quality of evidence.

## QUESTION 5

In patients with IBD, what is the effect on maintenance or discontinuation of treatment with biosimilar biologicals compared to the originator biologics after multiple switches?

### Practice point - Question 5

The experts have debated multiple switches between the originator molecule and its biosimilars. Although each biosimilar is shown to be highly similar to the originator biologic, such similarity cannot be presumed across different biosimilars; this limitation remains a central concern in discussions on multiple switching. Doubts are raised among prescribing physicians, as payers could perform multiple switches, potentially compromising patient access to biological therapy. The primary concern would be the induction of immunogenicity. The definition of “Multiple Switches” in the context of biologic treatment is arbitrary. Currently, multiple switches are defined when two or more changes occur across different versions of the same originator biologic product, including switches between biosimilar biologics. Evidence on this issue has emerged from real-world studies in which two or more biosimilars were switched between themselves and with the originator biologics.

Studies were published on multiple switches involving infliximab, comparing patients who underwent a single switch (from the originator IFX to IFX-CTP13) with those who underwent multiple switches (from the originator IFX to IFX-CTP13 and, subsequently, to SB2). Lovero et al.^63^ described a series of 36 patients with clinically stable IBD, of whom 24 underwent only one switch, and 12 underwent multiple switches. During follow-up of these patients receiving IFX-SB2, there was no difference in loss of response between those who underwent a single switch and those who underwent multiple switches. In a similar prospective study comparing single or multiple switches, Trystram et al. 48 again found no difference between the groups, a result also observed by Khan et al.^64^ and collaborators. In 2023, Gros et al.^49^ evaluated patients who underwent 1, 2, or 3 infliximab switches using the biosimilars SB2 and CTP-13, in addition to the originator. The number of switches performed was unrelated to the drug’s effectiveness after excluding confounding factors.

#### Evidence Synthesis Table 6 ^17^ ^,^ ^34^ ^,^ ^48^ ^,^ ^49^ ^,^ ^63^ ^-^ ^65^.

**TABLE 6:** Evidence Synthesis of question 5: In patients with IBD, what is the effect on maintenance or discontinuation of treatment with biosimilar biologics compared to the originator biologics after multiple switches?

First author	Year	Study design	N population	Evidence Synthesis
Lontai L	2022	Prospective multicenter	276	No statistically significant difference was found in the proportion of patients in clinical remission at week 8-12 prior switch / switch / week 8-12 and week 20-24 after switch in patients who were switched from biosimilar-to-biosimilar adalimumab treatment (74.5% / 78.4% / 85.3% / 79.8%; *P*=0.291 among groups)^17^.
Park J	2023	Randomized controlled trial (RCT)	198	Study arm (CT-P13-CT-P13, CT-P13-infliximab, infliximab-CT-P13, and infliximab-infliximab) was not associated with clinical remission at week 54 in univariable logistic regression analysis. There were no statistical differences in serum infliximab trough levels and antidrug antibodys levels between CT-P13 and originator infliximab^34^.
Trystram N	2021	Prospective cohort study	158	The probability of event-free survival after switching from CT-P13 to SB2 was 97.4%, 91.6%, 89.5% and 89.5% at 3, 6, 9 and 12 months after switching respectively. No difference was observed between the double and single switch groups (HR=0.36, 95%CI [0.09-1.42], *P*=0.14). The probability of treatment persistence after switching from CT-P13 to SB2 was 98.1%, 96.2%, 94.9% and 94.9% at 3, 6, 9 and 12 months after switching respectively. No difference was observed between the double and single switch groups (HR=1.87, 95%CI [0.70-11.25], *P*=0.46)^48^.
Gros B	2023	Prospective, observational, cohort study	297	Subgroup analysis based on number of IFX switches revealed that IFX persistence was 82.6%, 92.8% and 97% in patients with in respective one, two and three IFX switches (*P*=0.003). However the number of switches was not independently associated with IFX persistence after adjusting for confounders^49^.
Lovero R	2021	Retrospective cohort	36	After comparing patients with (n=11) or without (n=25) LOR, the latter subset had more often a previous double infliximab switch, but the difference was NS (40% versus 9.1%, *P*=0.06; OR=0.13; 95%CI 0.01-1.15)^63^.
Khan N	2024	Prospective cohort study	271	Within the single-switch group, 12.9% of patients discontinued SB2 within 1 year and 11.9% of patients continued SB2 but were not in disease remission after 1 year. Within the double-switch group, 17.6% of patients discontinued SB2 within 1 year and 9.4% of patients continued SB2 but were not in IBD remission after 1 year. There was no statistically significant difference observed in regard to efficacy or safety of the single switch versus double switch to SB2^64^.
Fitzgerald T	2021	Retrospective database	1109	Of the switchers who switched to another biologic originator, 87.7% switched back to IFX. Over a mean post-index observation period, 21.0% switchers and 7.1% continuers switched to another originator biologic (including originator IFX) (HR [95%CI]=3.42 [2.36, 4.94]; *P*<0.001)^65^.

#### Recommendations - Question 5

The number of switches was not independently associated with persistence in the biological treatment, nor was it related to clinical remission or loss of response. Therefore, although the evidence is limited, multiple switches cannot be deemed ineffective or unsafe. Due to the evidence’s low quality, it is not possible to recommend for or against multiple switches.

Low quality of evidence.

## QUESTION 6

In pediatric patients with IBD, what is the efficacy and safety of biosimilar biologics compared to originator biologics?

### Practice point - Question 6

The treatment of IBD in pediatric patients remains challenging, particularly due to the limited availability of approved biologic therapies for this age group. Available evidence, derived mainly from real-world studies and a limited number of trials, suggests that biosimilar biologics have efficacy and safety profiles comparable to those of originator biologics in children and adolescents with IBD.

Clinical evidence supports the comparability of the infliximab biosimilar CT-P13 with the originator biosimilar in pediatric patients with IBD. In a cohort of patients under 19 years, CT-P13 demonstrated similar endoscopic healing rates and adverse event profiles, including infusion reactions and hospitalizations, after one year of follow-up^66^. Growth parameters and disease control were comparable in a propensity-score matched study comparing CT-P13 and originator infliximab^30^, consistent with a review that included patients up to 21 years of age^67^.

Further confirmation arises from multiple pediatric studies showing equivalence in clinical outcomes for both infliximab and adalimumab biosimilars used as first-line therapy in biologicnaïve children and adolescents^68^ ^,^ ^69^ ^,^ ^70^. Moreover, a retrospective study observed no difference in drug persistence, corticosteroid-free remission, dose escalation, or serum infliximab concentrations one year after switching to CT-P13 from the originator^71^.

Several studies consistently show no statistically significant differences in clinical remission, mucosal healing, growth parameters, or drug retention between biosimilars and the originator. Kim et al.^66^: in a robust multicenter cohort of 416 pediatric IBD patients, both CT-P13 and IFX-O demonstrated similar clinical and endoscopic remission at 1 year, with comparable EH rates and treatment durability over >5 years. McClinchie et al.^30^: growth trajectories (weight, height, BMI z-scores) and disease activity improvement were parallel in both groups over 12 months. Kang et al.^71^: a switch from originator to biosimilar during maintenance therapy yielded no significant differences in trough levels, immunogenicity, or disease control.

Evidence regarding growth trajectories, immunogenicity, trough levels, and corticosteroid-free remission also appears comparable between biosimilars and originator biologics. However, estimates remain imprecise due to heterogeneity in study design and outcome definitions. Overall, current data do not indicate clinically meaningful differences in efficacy or safety outcomes between biosimilar and originator biologics in pediatric IBD. However, the evidence’s certainty is limited by the predominance of observational studies, small sample sizes, and the absence of randomized trials specifically designed for this population.

#### Evidence Synthesis Table 7 ^30^ ^,^ ^66^ ^-^ ^71^.

**TABLE 7:** Evidence Synthesis of question 6: In pediatric patients with IBD, what is the efficacy and safety of treatment with biosimilar biologics compared to the reference biologics?

First author	Year	Study design	N population	Evidence Synthesis
McClinchie MG	2023	Retrospective cohort	152	Similar improvements in growth and clinical outcomes were observed in children initiated on the infliximab originator compared with an infliximab biosimilar^30^.
Kim ES	2024	Retrospective cohort	416	The infliximab originator and its biosimilar CT-P13 exhibited comparable therapeutic responses, including EH and clinical remission, durability, and safety in pediatric patients with IBD. Our results are significant in that this is the first study to directly compare clinical outcomes, including EH at 1 year after treatment in pediatric patients with IBD treated with an IFX originator and CT-P13^66^.
Hinshaw A	2022	Retrospective cohort	25	When comparing biochemical markers of disease activity, Infliximab biosimilar is as effective as Infliximab originator for maintenance therapy in pediatric inflammatory bowel disease^67^.
Chanchlani N	2018	Prospective cohort	676	Infliximab biosimilar is likely as effective as infliximab orinator in treating IBD in comparable pediatric populations^68^.
Nikkonen A	2020	Retrospective cohort	51	In the treatment of children with IBD, the first-year therapy outcomes of the infliximab originator and its biosimilar were comparable. There were no alarming signs of differences in safety^69^.
O’Connell DM	2022	Retrospective cohort	56	An infliximab biosimilar appears safe in children, with no increased infusion reactions, and reduces costs^70^.
Kang B	2018	Prospective observational	74	Switching from maintenance infliximab originator to biosimilar CT-P13 did not result in significant differences in efficacy, pharmacokinetics, or immunogenicity in patients with pediatric-onset IBD, and no unexpected safety issues occurred^71^.

#### Recommendations - Question 6

In pediatric patients with IBD, the efficacy and safety of biosimilar biologics compared to originator biologics appear similar. Still, to date, there has been no quantification, estimated or precise.

Very low quality of evidence.

## QUESTION 7

In patients with IBD, what is the impact on the cost of treatment with biosimilar biologics compared to the originator biologics?

### Practice point - Question 7

The NORTH^38^ study, a Swedish observational cohort, evaluated the total cost of patients who switched from the originator to a biosimilar versus those who did not, using propensity score matching. At the end of follow-up, the total cost was approximately 4 times higher among patients who continued the originator molecule than among those who switched (€16,740 vs €3,872; *P*=0.0000). On the other hand, among those who switched, the mean total medical cost was higher in the year following the switch compared to the year prior (€1,452 vs €802; *P*<0.0196).

Another study assessed the annual cost per patient with ulcerative colitis, biologic-naïve, treated with either the infliximab originator (€6,140.44) or its biosimilar (€1,713.29), demonstrating a significant cost reduction^35^. These findings are supported by Plevris et al.^72^, who reported a 46.4% cost savings in patients with Crohn’s disease treated with a biosimilar. Another study by Severs et al.^73^ found that a 5-year projection showed that biosimilars could cut IBD costs by €493 million, representing a 28% reduction. Per-Patient Savings: €9,850 (CD) and €2,250 (UC).

The use of biosimilars has been associated with reduced costs without compromising efficacy or negatively impacting patient-reported outcomes and quality of life. The available real-world evidence suggests that incorporating anti-TNF biosimilars into IBD treatment strategies can result in significant cost savings without compromising safety or efficacy.

Switching from originator infliximab (IFX) to biosimilars results in substantial cost savings without compromising clinical outcomes, making biosimilars a cost-effective strategy for treating IBD.

#### Evidence Synthesis Table 8 ^35^ ^,^ ^38^ ^,^ ^70^ ^,^ ^72^ ^-^ ^79^.

**TABLE 8:** Evidence Synthesis of question 7: In patients with IBD, what is the impact on the cost of treatment with biosimilar biologics compared to the originator biologics?

First author	Year	Study design	N population	Evidence synthesis
Valcuende-Rosique A	2022	Observational comparative study	80	In biologic-naïve ulcerative colitis patients, annual per-patient costs were markedly lower with biosimilar infliximab compared with the originator (€1,713.29 vs €6,140.44). Switching all originator-treated patients to biosimilars would have generated annual hospital savings exceeding €200,000^35^.
Hellström PM	2022	Observational cohort (propensity score-matched)	222	In the Project NORTH study, total medical costs were significantly higher in non-switchers than in patients who switched from originator infliximab to a biosimilar (€16,740 vs €3,872; P<0.001). Among switchers, mean total medical costs increased in the year after the switch compared to the year before (€1,452 vs €802; P<0.02), but remained substantially lower than in non-switchers^38^.
O’Connell DM	2022	Retrospective cohort with cost analysis	NA	Use of an infliximab biosimilar resulted in mean savings of US$2,611 per infusion. Rapid infusion protocols further reduced infusion time, nursing workload, and infusion center occupancy, contributing to additional healthcare resource savings^70^.
Plevris N	2019	Prospective service evaluation	110	Switching from originator infliximab to CT-P13 resulted in approximately 46.4% cost savings, with no negative impact on clinical outcomes, drug levels, or safety over 12 months^72^.
Severs M	2017	Economic modeling study	NA	A 5-year projection model estimated that the introduction of biosimilar infliximab could reduce national IBD healthcare costs by 28%, corresponding to total savings of approximately €493 million, with per-patient savings of €9,850 (CD) and €2,250 (UC)^73^.
Morris GA	2022	Quality improvement intervention	NA	Increasing biosimilar utilization from 1% to 96% was associated with substantial cost savings (approximately US$381,000 based on average sales price and US$651,000 based on wholesale acquisition cost), without compromising clinical outcomes or safety^74^.
Hughes A	2020	Cost-utility analysis (Markov model)	NA	Switching from originator to biosimilar infliximab was associated with incremental cost savings of US$46,194 but a slight loss in QALYs (-0.13). Most simulations favored cost savings, although decision-makers must weigh reduced costs against minimal effectiveness loss.
Phisalprapa P	2020	Cost-effectiveness and budget impact analysis	NA	The infliximab biosimilar was not cost-effective at current prices in patients with refractory Crohn’s disease. Cost-effectiveness would require a price reduction of 72-90%. Nevertheless, biosimilar strategies demonstrated lower ICERs and a more favorable budget impact than originator infliximab^76^.
Huoponen S	2020	Prospective observational study	54	Switching from originator to biosimilar infliximab reduced drug acquisition costs to approximately one-third of the originator cost. In contrast, health-related quality of life, disease activity, and non-drug healthcare costs remained comparable during 12-month follow-up^77^.
Razanskaite V	2017	Observational service evaluation	143	A managed switching program from originator infliximab to CT-P13 reduced drug acquisition costs by £40,000-60,000 per month, while maintaining similar patient-reported outcomes, drug persistence, and safety^78^.
Brodszky V	2014	Budget impact analysis (economic model)	NA (population-based model)	This budget impact analysis evaluated patients with Crohn’s disease treated with biologics across six Central and Eastern European countries (Bulgaria, Czech Republic, Hungary, Poland, Romania, and Slovakia). Gradual interchange of originator infliximab with the biosimilar infliximab was projected to yield substantial cost savings over 3 years (€16.6 million) or expand access to biologic therapy for hundreds of additional patients, depending on switching policies^79^.

#### Recommendations - Question 7

Where feasible, health systems should implement a structured, gain-sharing-enabled switch from the originator biologic to a biosimilar in IBD to realize cost savings and reinvest in care while maintaining comparable clinical outcomes and safety and reducing the risk of a nocebo effect.

Very low quality of evidence.

## QUESTION 8

In patients with IBD, what is the effect on mucosal healing of treatment with biosimilar biologics compared to the originator biologics?

### Practice point - Question 8

Mucosal healing is a key therapeutic goal. Multiple studies, both observational and randomized, consistently demonstrate that biosimilar anti-TNF agents are effective in inducing and maintaining clinical and endoscopic remission in patients with IBD. The study by Kim et al.^66^. offers crucial real-world evidence on the effectiveness of biosimilar infliximab (CT-P13) compared to the originator infliximab (IFX) in promoting mucosal healing in this population. The study found that mucosal healing rates were comparable between the two groups. Endoscopic assessments performed on clinical indications demonstrated similar levels of mucosal improvement, indicating that CT-P13 is as effective as originator IFX in both inducing and maintaining mucosal healing.

Three studies^20^ ^,^ ^34^ ^,^ ^36^ demonstrated that switching from originator to biosimilars (IFX or ADL) maintained clinical and endoscopic remission, with no significant increase in relapse or loss of response. These data, which demonstrate equivalence in mucosal healing outcomes, reinforce biosimilars as a viable and cost-effective therapy for treating patients with IBD.

Evidence Synthesis Table 9 ^14^ ^,^ ^20^ ^,^ ^24^ ^,^ ^34^ ^,^ ^36^ ^,^ ^40^ ^,^ ^53^ ^,^ ^56^ ^,^ ^66^.

**TABLE 9:** Evidence Synthesis of question 8: In patients with IBD, what is the effect on mucosal healing of treatment with biosimilar biologics compared to the originator biologics?

First author	Year	Study design	N population	Evidence Synthesis
Chaparro M	2019	Retrospective, cohort	476	Switching from original infliximab to infliximab CT-P13 might be associated with a higher risk of clinical relapse, although objective markers of inflammation do not support this fact^14^.
Casanova MJ	2023	retrospective cohort	524	Switching to adalimumab biosimilar did not impair patients’ outcomes, such as therapy discontinuation, relapse, or adverse events, compared to maintaining the originator^20^.
Farkas K	2016	Prospective observational cohort	47	In patients with moderate-to-severe ulcerative colitis receiving induction therapy with biosimilar infliximab CT-P13, mucosal healing rates were high and comparable to historical data with originator infliximab. Clinical response and remission were achieved without new safety signals, supporting the efficacy of CT-P13 for induction therapy in UC^24^.
Park J	2023	Randomized controlled trial	198	A threshold in serum infliximab trough level at weeks 6 and 14 was highly predictive of long-term clinical outcomes^34^.
Wetwittayakhlang P	2024	Prospective observational	210	Despite a significant number of early nocebo complaints within the first 6 months after the biosimilar switch, no significant changes were found in clinical efficacy, biomarkers, therapeutic drug level, or anti-drug antibodies^36^.
Barberio B	2020	Retrospective Cohort	179	At week 52, 31.1% of adalimumab originator, 16.7% of IFX originator, and 36.2% of biosimilar patients needed treatment optimization^40^.
Mocci G	2022	Retrospective Cohort	134	This real-world data analysis suggests that adalimumab GP2017 (biosimilar) and its originator are equivalent in terms of efficacy and safety in patients with IBD^53^.
Ye BD	2019	Randomized Controled Trial	220	In patients with active Crohn’s disease, infliximab CT-P13 was non-inferior to the originator infliximab in terms of clinical efficacy and safety over 54 weeks^56^.
Kim ES	2024	Retrospective cohort study	252	In pediatric patients with inflammatory bowel disease treated with infliximab originator or biosimilar CT-P13, rates of endoscopic healing and long-term treatment durability were comparable between groups. No significant differences were observed in sustained response, loss of response, or need for treatment escalation, supporting comparable effectiveness of CT-P13 in the pediatric population^66^.

#### Recommendations - Question 8

In patients with IBD, available evidence suggests that mucosal healing rates with biosimilar biologics are comparable to those achieved with originator biologics, with no relevant differences between switch and non-switch groups.

Low quality of evidence.

## CONCLUSION

This expert consensus, developed through a structured systematic review and Delphi methodology, provides evidence-based guidance on the use of biosimilar biologics in IBD. Overall, the evidence suggests that anti-TNF biosimilars have efficacy, safety, immunogenicity, treatment persistence, and mucosal healing outcomes comparable to those of originator biologics, both in biologic-naïve patients and after switching.

Although the quality of the evidence is predominantly low to very low, mainly due to the observational design of most studies, findings are consistent across randomized trials, real-world cohorts, and economic evaluations. Importantly, current data do not indicate clinically meaningful differences in adverse events, loss of response, or treatment discontinuation between biosimilars and originator products. Evidence regarding multiple switching also does not suggest a negative impact on clinical outcomes, although the available data remains insufficient to support definitive recommendations.

In pediatric patients, available studies indicate that biosimilars may offer efficacy and safety profiles similar to those of originator biologics, albeit with limited precision. At the same time, economic analyses consistently indicate substantial cost savings, supporting the potential role of biosimilars in improving access to biologic therapy and contributing to healthcare system sustainability.

In summary, biosimilars represent a viable, safe, and cost-effective therapeutic option for the management of IBD. Structured switching strategies, appropriate patient communication, and ongoing clinical monitoring should support their implementation. Further high-quality, methodologically robust studies are needed to strengthen the evidence base, particularly in specific populations and clinical scenarios.

## Data Availability

not applicable - the study did not use research data
